# Co-design of a novel rehabilitation intervention for patients after ankle fracture surgery: establishing healthcare professional consensus

**DOI:** 10.1007/s12306-025-00881-1

**Published:** 2025-01-23

**Authors:** C. Bretherton, A. Al-Saadawi, P. H. Sandhu, P. J Baird, P. X. Griffin

**Affiliations:** 1 Bone and Joint Health, Blizard Institute, Queen Mary University London, 4 Newark Street, London, E1 2AT UK; 2 Department of Trauma and Orthopaedic Surgery, Royal London Hospital, Barts Health NHS Trust, London, E1 1BB UK; 3 School of Medicine, Faculty of Medicine and Dentistry, Queen Mary University of London, London, UK; 4 Division of Health Sciences, Warwick Clinical Trials Unit, University of Warwick, Coventry, CV4 7AL UK; 5 Centre for Developmental Origins of Health and Disease, University of Southampton, Southampton, SO17 1BJ UK

**Keywords:** Ankle fracture, Rehabilitation, Behaviour change wheel, Consensus, Digital health

## Abstract

Post-surgical rehabilitation advice after ankle fracture surgery, particularly regarding weight-bearing, varies significantly, leading to patient frustration and inconsistent recovery outcomes. This study aimed to establish a consensus for ankle fracture rehabilitation advice and identify content and implementation options for future interventions through consultation with healthcare professionals (HCPs). This study was part of the weight-bearing in ankle fractures (WAX) trial, a multicentre, randomised controlled trial. Using the behaviour change wheel (BCW) framework, three online workshops with HCPs were conducted between April 25, 2022, to May 4, 2022, to generate consensus on rehabilitation interventions. Participants completed pre-workshop tasks, and data were collected using an adapted nominal group technique (NGT). Workshop data were collated to create a survey with indicative statements about rehabilitation preferences. An online survey was subsequently disseminated to surgeons and physiotherapists between May 5, 2022, and July 13, 2022. Respondents were asked to indicate how strongly they agreed with various statements by ranking statements on a 5-point Likert scale from "strongly disagree" to "strongly agree”; 75% was used as a threshold for consensus agreement. Eight HCPs participated in the workshops, and 79 HCPs responded to the survey, representing 38 different NHS hospital trusts. Consensus was achieved on several key aspects: Patients could rest their foot on the floor while seated during non-weight-bearing periods and gradually increase weight-bearing based on comfort. It was agreed that orthotic boots are for comfort, and patients can discontinue use as early as two weeks post-operatively if weight-bearing is permitted. Guidelines for wound management, including when to get wounds wet and how to handle numbness, were also established. This study established a consensus for ankle fracture rehabilitation, emphasising patient autonomy and clear, standardised advice. The findings support the development of a tailored, patient-centred rehabilitation interventions, potentially delivered through digital platforms, to enhance recovery outcomes.

## Introduction

### Background

Ankle fractures are prevalent in the UK, accounting for approximately 70,000 cases annually [1]. These fractures can be managed conservatively or surgically, with unstable fractures necessitating surgical fixation to correct the deformity [2, 3]. There exists significant variability in post-surgical rehabilitation advice offered to patients, particularly regarding weight-bearing [2]. Patients express frustration that available patient-facing ankle fracture literature does not provide more specific advice, such as when to discontinue orthotic boot use or start bathing. This is because there is no consensus around these topics, and advice for one patient, could be potentially unsafe for another. New evidence around fixation techniques, orthotic boot use and metalwork removal has begun to shift practice [4–7]. Now is an appropriate time to seek consensus around some of these topics for patient-facing advice so that patients are no longer faced with uncertain, variable advice which may delay their recovery and, in the long-term, potentially cause chronic ankle instability [8].

Co-design or co-production entails a collaborative effort between clinicians, researchers and the public to deliver research and design interventions in a holistic manner. [9, 10]. The behaviour change wheel (BCW) is a co-design model used to describe, design and evaluate interventions through the application of 19 behavioural change frameworks [11]. It consists of three core stages: (1) understand the behaviour, (2) understand the intervention options and (3) understand the content and implementation options. After identifying the behavioural problem, phases two and three of the BCW utilise a qualitative approach, consisting of interviews and workshops, to gain deeper insight into the nature of the behavioural problem and develop effective solutions with stakeholders [12–14]. Using the BCW approach, the objectives of this research were to establish a consensus for advice for ankle fracture rehabilitation and identify content and implementation options for future interventions through consultation with healthcare professionals. In particular, the research aimed to establish consensus around timeframes for commencing rehabilitation and explore novel options for service provision and patient follow-up.

## Methods

### Study design

This study was incorporated within the weight-bearing in ankle fractures (WAX) trial, published separately [15]. WAX was a multicentre, randomised controlled trial conducted in the UK that aimed to study early weight-bearing following operatively managed ankle fractures compared to traditional delayed weight-bearing regimens. A protocol for the WAX trial was registered on the 2nd of December 2019 (ISRCTN12883981) and has been published [16]. The South Central Oxford A Research Committee granted ethical approval for this study on the 22nd of November 2019 (Reference: 19/SC/0566).

A rehabilitation strategy was co-designed using the three phases of the BCW [11]. In phases one and two, a list of candidate behaviour change intervention bundles was generated [17] using responses from previous qualitative interviews, workshops and consensus surveys from patients recovering from ankle fractures. Phase three (understand the content and implementation options) is the subject of this paper.

**Fig. 1:**
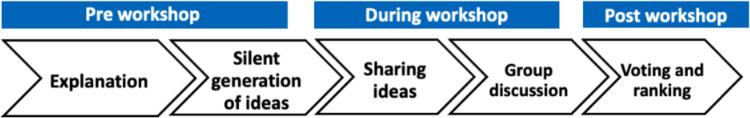
Online nominal group technique (NGT)

1. Understand the behaviour (conducted prior)
  This was done by assessing participants’ capability, opportunity and motivation to engage with the behaviour (weight-bearing and progressing rehabilitation) via qualitative interviews which have been published separately [18, 19].
2. Understand the intervention options (conducted prior)
  Key barriers, enablers and areas needing change as identified during the qualitative interview analysis were mapped the nine intervention functions of the BCW [20]. A list of candidate behaviour change intervention bundles were then generated [17]. The behaviour change interventions were refined and prioritised during workshops and consensus surveys with patients recovering from ankle fractures.
3. Understand the content and implementation options
  This was done using the affordability, practicability, effectiveness/cost-effectiveness, acceptability, safety/side effects (APEASE) criteria [11]. An online nominal group technique (NGT), adapted from Bateman et al. [21] and Fisher et al. [22], was used and reported in accordance with the checklist for reporting of survey studies (CROSS) [23]. Figure 1 outlines the stages of the NGT.

### Setting and sampling

Healthcare professional (HCP) participants were recruited through existing professional networks and the WAX trial. They were contacted by email and provided with a participant information sheet. The workshops were conducted virtually, and HCPs completed an online consent form before the workshop.

A purposive maximum variation sampling strategy was used to obtain a spread of staff treating patients with ankle fractures in different roles and regions. Three groups were sought: 1) physiotherapists, (2) consultant trauma and orthopaedic surgeons and 3) trainee trauma and orthopaedic surgeons. These groups were chosen as the key decision-makers for patients recovering from ankle fractures.

### Data collection methods

Three two-hour workshops took place on April 25th, April 29th, and May 4th, 2022, facilitated by the lead author (CB). Based on the same maximum variation sampling strategy described above, two or three HCPs attended each workshop in addition to a patient representative. All participants completed pre-workshop tasks, including watching an explanatory video and completing a worksheet. The purpose was to determine the content and phrasing of a consensus survey for surgeons and physiotherapists. A series of statements around different aspects of ankle fracture surgery were iteratively refined and updated. All workshops groups were recorded. After the workshop, all recordings were reviewed, and the statements and survey were iteratively updated, ready for the next workshop.

### Survey

An online survey was compiled using data from the workshop. The REDCap survey link was emailed to all surgeons and physiotherapists on the WAX trial delegation log as well as disseminated via social media. A £10 shopping voucher was offered for survey completion. The survey was open from May 5th—July 13th 2022.

An online video and introduction page explained the purpose of the survey and how to complete it. Respondents were told that the survey aimed to create standardised concepts or phrases that can be given to patients to aid their recovery. The aim was to create advice that > 75% of HCPs would be happy to provide to > 75% of their patients post-operatively. It was explained that if their patient was in the other 25%, their situation would somehow be atypical, and they would not be offered a generic rehabilitation programme.

Respondents were asked to indicate how strongly they agreed with various statements by ranking statements on a 5-point Likert scale from "strongly disagree" to "strongly agree". There was also an option to select "I cannot comment in my role", as some questions were directed at specific groups of HCPs, and it was anticipated that some physiotherapists would not be comfortable making decisions for certain aspects of care (e.g. surgical decision-making). At the end of each section, a space for free-text comments was provided.

### Analysis

Analysis was conducted in R, and figures were compiled using the package Likert [24, 25]. 75% was used as a threshold for consensus as it has been the most frequently employed threshold in published Delphi studies [26, 27], including those in orthopaedics [28, 29].

## Results

### Workshop and survey participants

Sixteen HCPs were invited to the workshops, of which eight (50%) agreed and attended. Participants included three physiotherapists and five trauma and orthopaedic surgeons (of which two were consultants and three were pre-consultant grades); sites and characteristics are shown in Appendix 1. The survey was emailed to 177 HCPs on the WAX trial delegation log, including those HCPs that attended the workshops. A total of 79 HCPs responded from 38 different hospital trusts, but as there was also an open invitation via social media, a formal response rate was not calculated. 53 (67%) responses were from trauma and orthopaedic surgeons and 26 (33%) from physiotherapists: Further characteristics of survey respondents are provided in Table 1 and in Appendix 2.

**Table 1:** Survey participant characteristics

Characteristic	N = 79
Job Role	
Trauma and orthopaedic surgeon	53 (67%)
Physiotherapist	26 (33%)
Surgeon grade	
Registrar, fellow or trust grade equivalent to ST3 +	32 (60%)
Consultant	21 (40%)
Consultant subspecialty	
Foot and ankle	9 (43%)
Trauma	7 (33%)
Other	5 (24%)
Physiotherapist primary role	
Inpatients	12 (46%)
Outpatients	14 (54%)

**Fig. 2:**
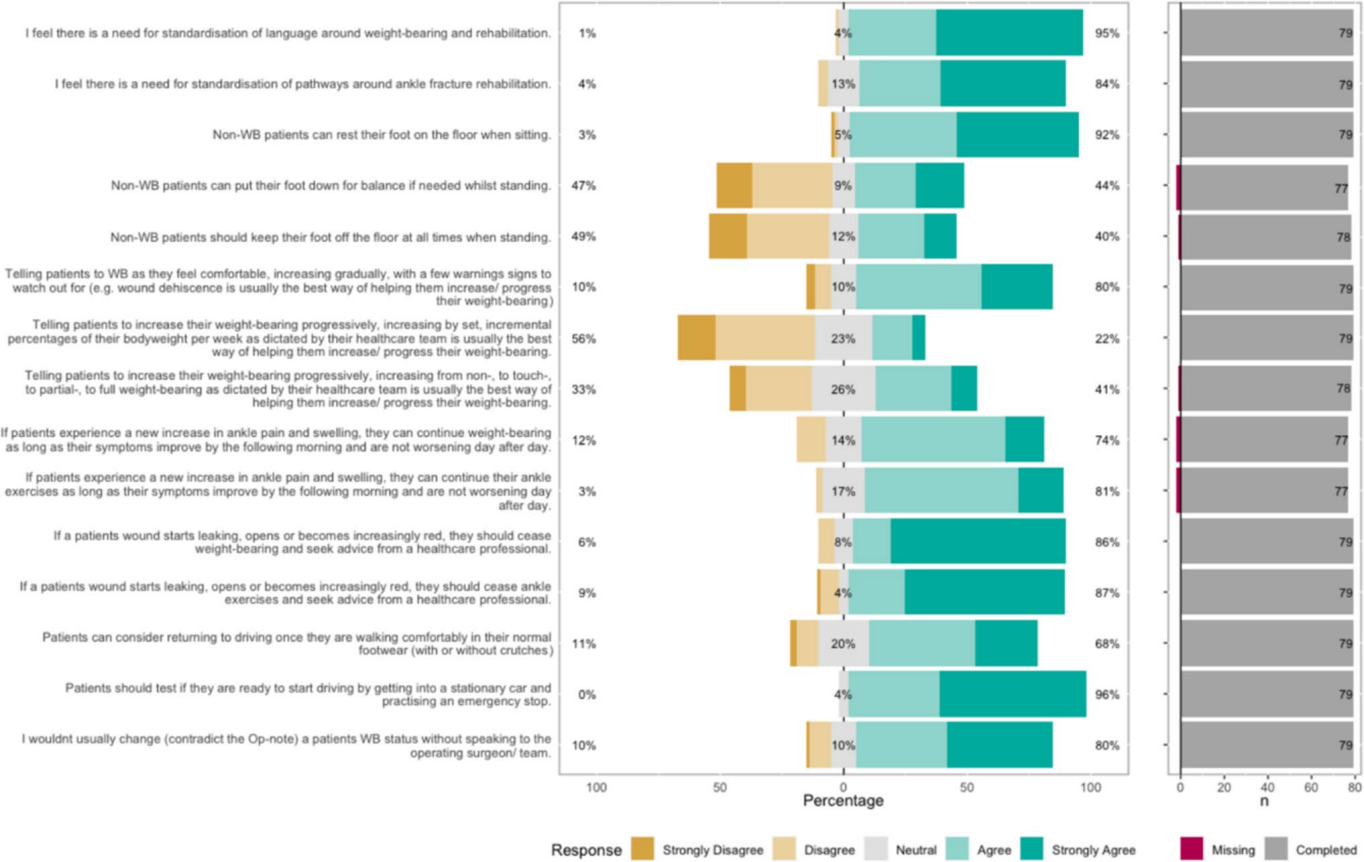
HCP Survey—Weight-bearing

**Fig. 3:**
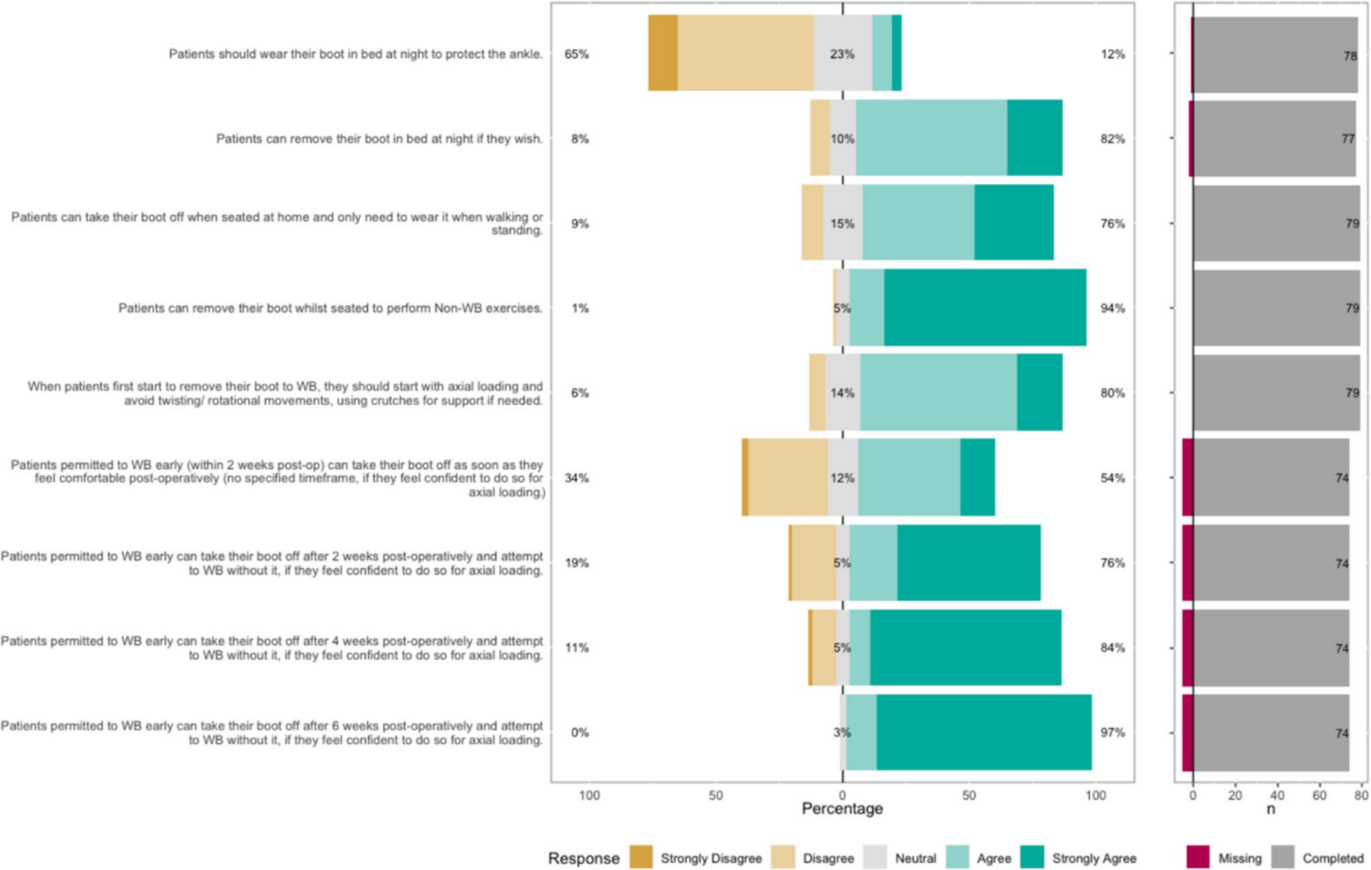
HCP Survey—Orthotic boot use

**Fig. 4:**
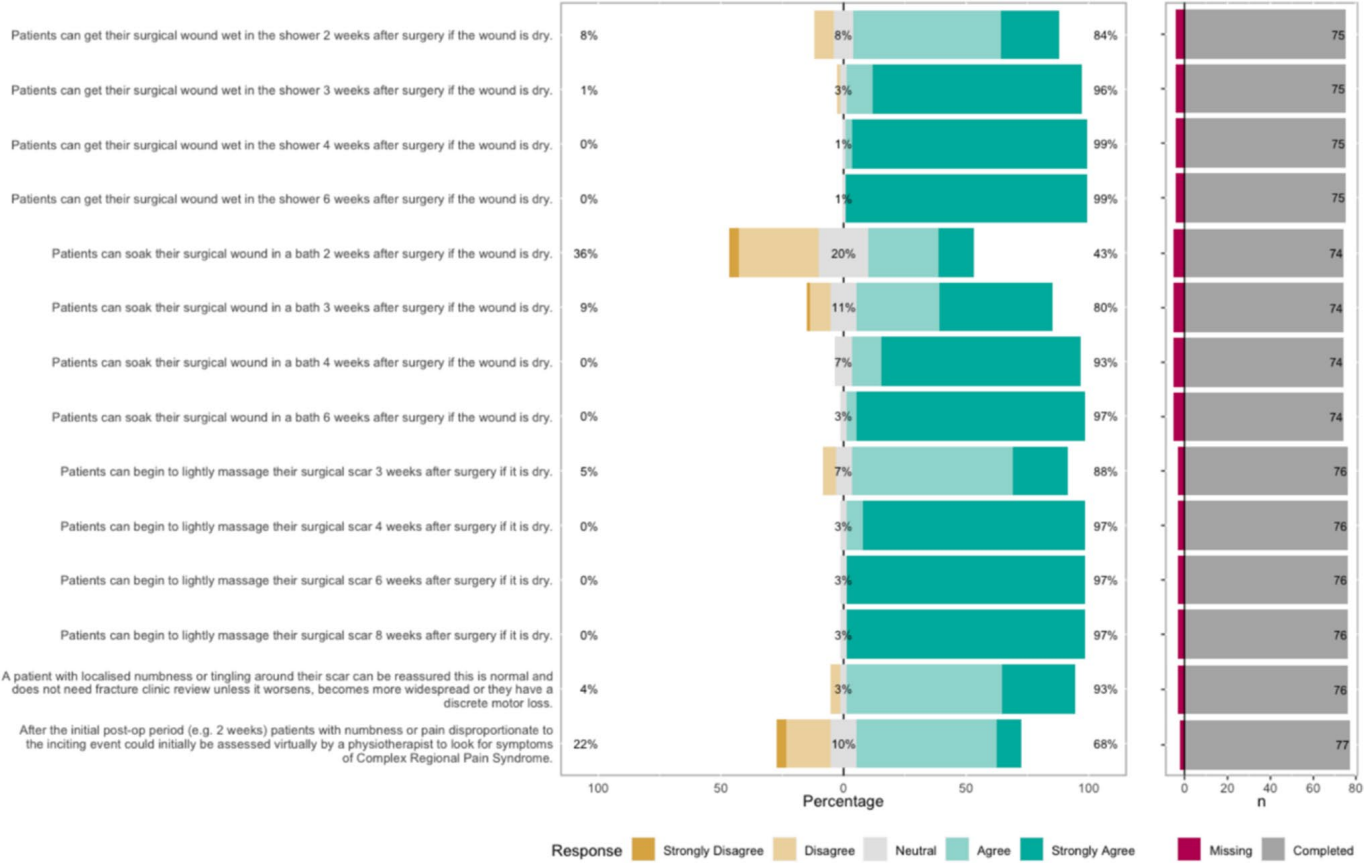
HCP Survey—Wound management

**Fig. 5:**
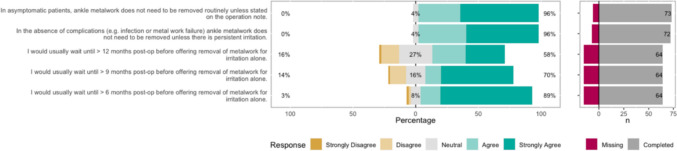
HCP Survey—Metalwork removal

**Fig. 6:**
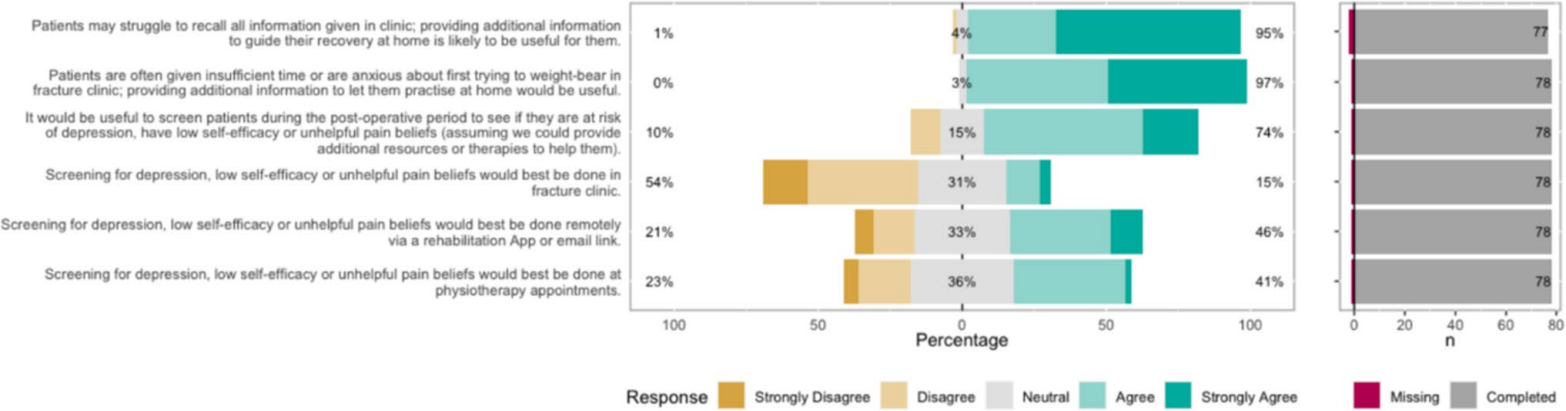
HCP Survey—Additional support

**Fig. 7:**
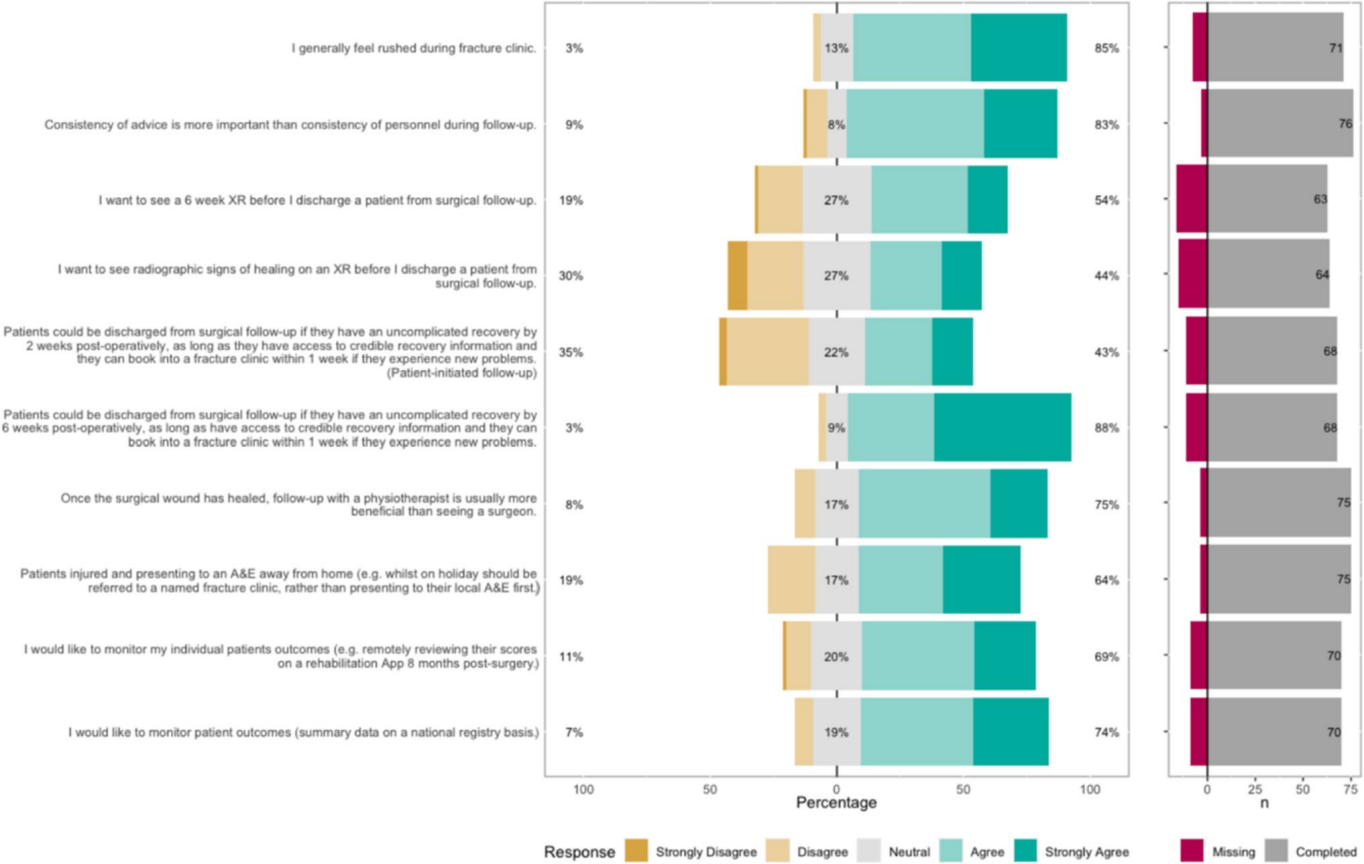
HCP Survey—Follow-up

**Fig. 8:**
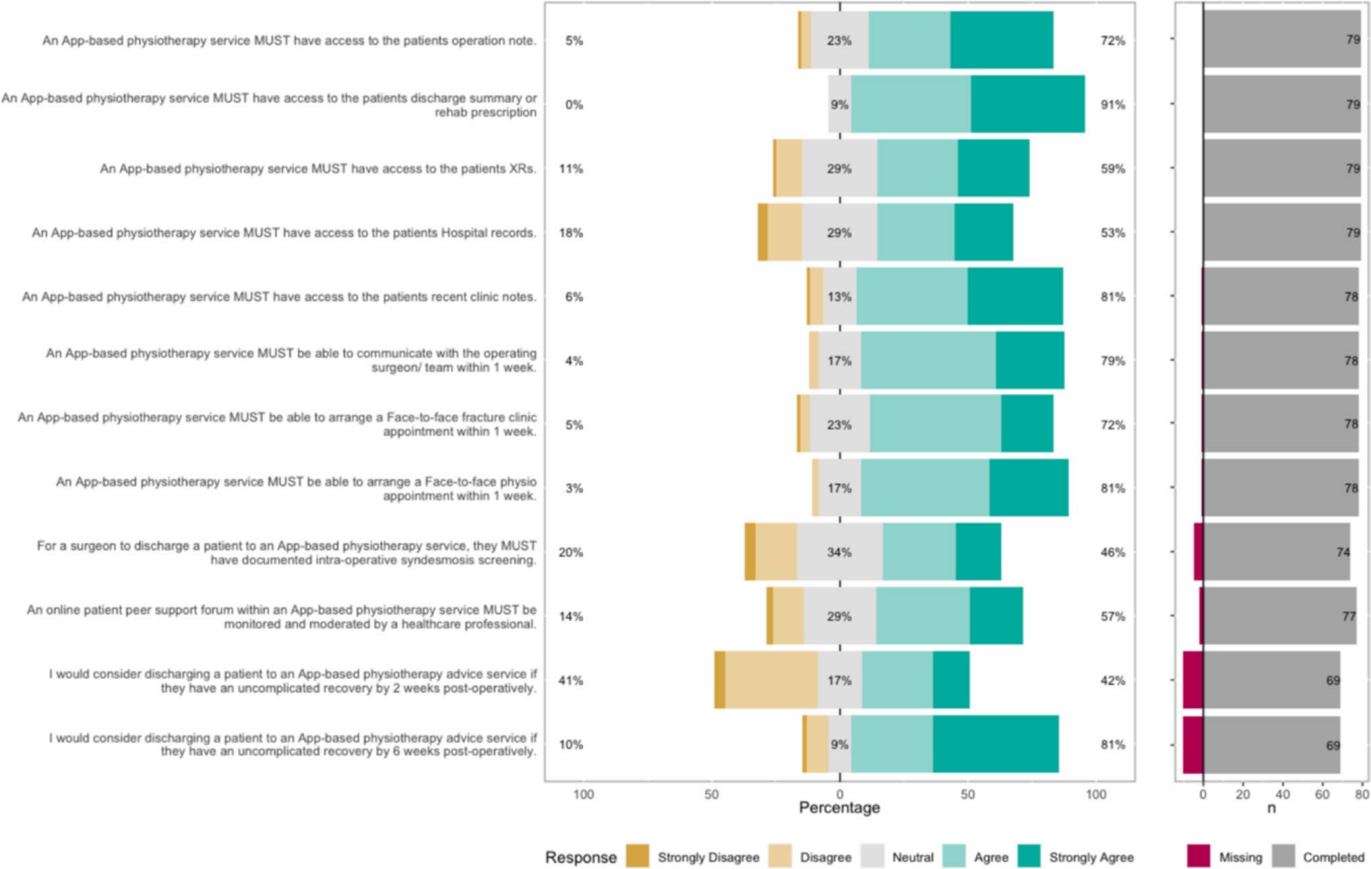
HCP Survey—An app-based physiotherapy advice service

**Fig. 9:**
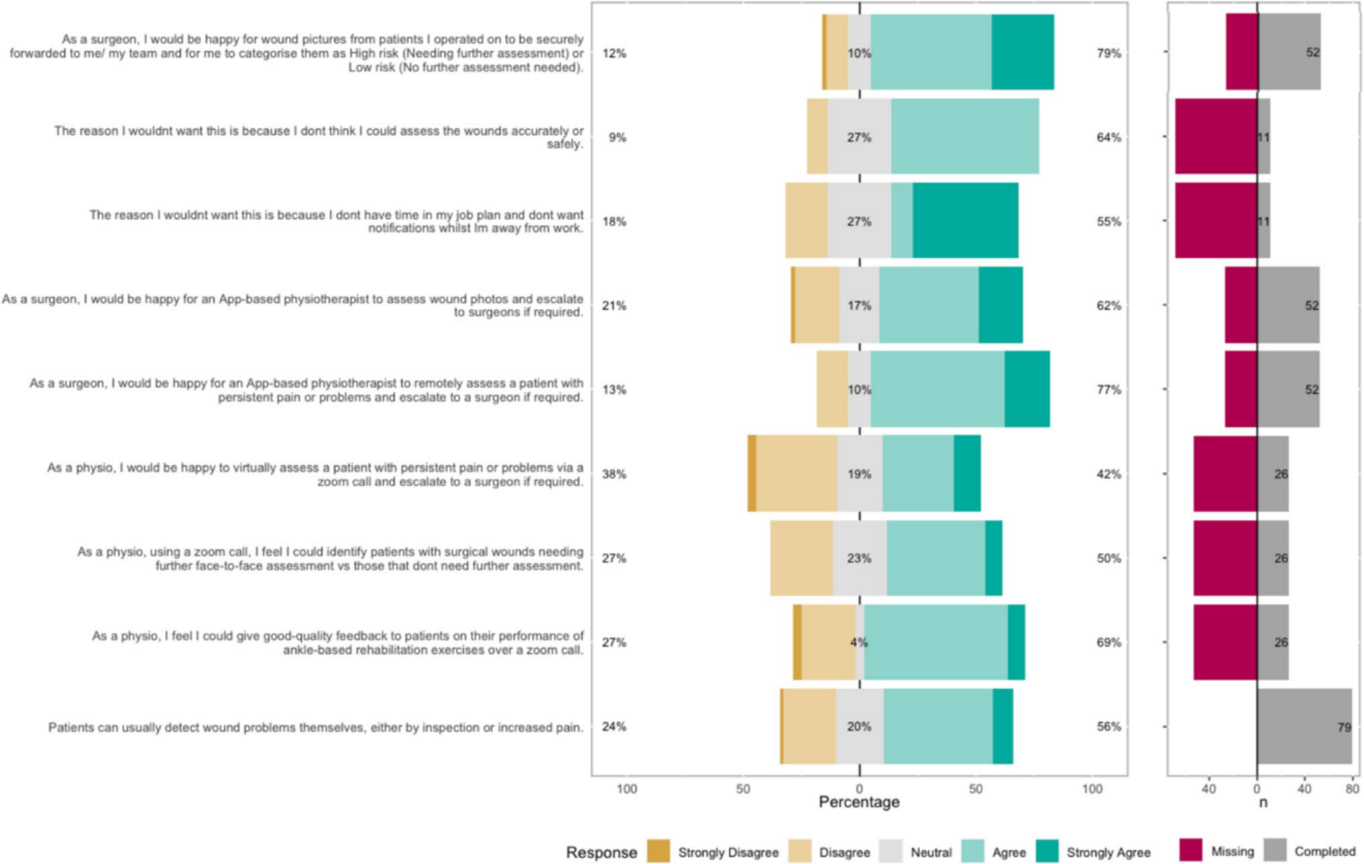
HCP Survey—Virtual follow-up

The Likert bar plots are displayed in Figs. 2, 3, 4, 5, 6, 7, 8 and 9. The percentages on the right side of the Likert bar plots correspond to the proportion of responses that were ranked "agree" or "strongly agree". The percentages on the left correspond to those ranked "disagree" or "strongly disagree", and the middle grey percentages to "neutral". Where responses are labelled as "missing", this relates to respondents selecting that they "cannot comment in [their] role", all questions mandated a response.

### Weight-bearing

Figure 2 shows the survey results for the weight-bearing (WB) statements. There was consensus that there is a need for standardisation of language and pathways around WB and rehabilitation. The survey attempted to find consensus for activities permitted during non-weight-bearing periods. HCPs agreed that patients could rest their foot on the floor while sitting during non-weight-bearing periods, but not for whether they could rest their foot down for balance while standing. HCP were in agreement that the best way to help patients increase and progress their weight-bearing was not to increase by set, defined amounts dictated by HCPs, but to increase gradually as their comfort allowed. A caveat was that giving patients warning signs to look out for (such as wound breakdown or redness) would be helpful. There was a consensus that HCPs would not usually contradict the operating surgeon’s weight-bearing instructions without discussing with them first.

With regard to when post-operative pain or swelling would be cause for alarm, the survey results indicated that as long as new pain or swelling settles by the following morning and does not worsen day after day, patients can continue weight-bearing and ankle exercises. Conversely, there was a consensus that if the wound starts leaking, opens or becomes red, patients should stop weight-bearing and exercises and seek advice from a HCP. The only consensus reached for driving was that patients should test if they are ready to start driving by getting into a stationary car and practising an emergency stop. (Consensus was not achieved for a specific timeframe.)

### Orthotic boot use

Figure 3 shows the survey results for the orthotic boot statements. A consensus was established that patients do not need to wear their boot in bed at night if they do not want to. Additionally, there was a consensus that patients need only wear their boot when they are actually walking and can remove it when seated or to perform exercises, even during their non-weight-bearing period.

There was a consensus that when first starting to weight-bear without their boot, patients should do so gradually and avoid rotational movements to begin with. The survey found that assuming patients had been permitted to weight-bear early, they could start trying to do so without their boot any time from two weeks post-operatively, within the caveats of the findings above.

### Wound management

Figure 4 shows the survey results for the wound management statements. Consensus was established, with HCPs indicating that patients could get their wound wet in the shower from two weeks post-operatively, and begin to soak and lightly massage their wound from three weeks post-operatively. This was with the caveat that the wound must be dry at this stage. The word "dry" was used instead of the word "healed", as this was a source of uncertainty found in previous interviews with patients, and dry was found to be acceptable to HCPs and understandable to patients.

Regarding patients concerns about numbness or tingling around their wound, HCP consensus was achieved that as long as this did not become widespread or have a discrete motor loss, patients could be reassured this was normal and did not need HCP review. However, HCPs had uncertainty when numbness, tingling or burning sensations would herald complex regional pain syndrome (CRPS) and consensus recommendations were not achieved.

### Metalwork removal

Figure 5 shows the survey results for the metalwork removal statements. There was a consensus that metalwork does not need to be removed routinely if patients are asymptomatic. Respondents were asked how long they would usually wait before offering removal of metalwork for irritation alone; 89% and 70% would wait at least six or nine months, respectively, implying the consensus level is around the eight-to-nine-month period. This does not mean this is when surgeons would be happy to remove the metalwork, but rather there is limited value in a patient presenting for assessment and requesting removal before then, as the majority of surgeons would not offer it.

### Additional support

Figure 6 shows the survey results for the additional support statements. There was a consensus that patients may face information overload and feel anxious about first attempting weight-bearing in clinic and that providing additional support at home would be helpful. Seventy-four per cent of respondents felt that screening for depression, low self-efficacy or unhelpful pain beliefs would be useful, but there was no agreement on when or where this should be done. Only 15% felt that fracture clinic would be the best place to perform this screening, with physiotherapy appointments or a remote app being considered a better place to do this.

### Follow-up

Figure 7 shows the survey results for the follow-up statements. There was a consensus that HCPs feel rushed during fracture clinic and that consistency of advice is more important than consistency of personnel during follow-up. Reducing unnecessary surgical follow-up was seen as a potential method for alleviating fracture clinic resources.

The survey asked respondents if they would consider discharging patients from clinic sooner than usual if the patient had access to credible recovery information and could book back into clinic quickly if they had problems. There was a consensus that HCPs would consider discharging patients with these provisions at six weeks, but only 43% of HCPs would consider this at two weeks post-operatively. Almost three-quarters of HCPs would like to review their aggregated patient outcomes via a national registry, though less wanted the ability to review their individual patient’s outcomes remotely.

### An app-based physiotherapy advice service

An app-based physiotherapy advice service has been proposed to enhance patient rehabilitation and potentially decrease the requirement for F2F follow-up. The HCP survey sought to define the logistic requirements for such a service. Figure 8 shows the survey results for the app-based physiotherapy statements. The notes that accompanied the survey explained that an app-based physiotherapy advice service would involve access to a physiotherapist via an online chat service from 9 am to 5 pm, Monday to Friday (as discussed in the workshops) and with the assumption that relevant patient records could be shared in a secure, encrypted form. Within these confines, there was a consensus that such a service should have access to the patient’s discharge summary or rehabilitation prescription and recent clinic notes. There was a consensus that a physiotherapist providing this service should be able to communicate with the responsible surgical team within one week to seek advice or clarification. Additionally, 81% felt that patients should be able to be offered a F2F physiotherapy appointment within one week to assess problems or if virtual sessions were inadequate. As with the findings in the follow-up section, over 80% of HCPs would consider discharging patients to an app-based physiotherapy service at six weeks, but only 42% would consider this at two weeks post-operatively.

### Virtual follow-up

Figure 9 shows the survey results for the virtual follow-up statements. There was a consensus among surgeons that they would be happy for wound pictures from their patients to be sent for them to assess remotely. However, during the workshops, some felt this would not be suitable. Results from the survey indicated that of the 11 surgeons that felt this was unsuitable, 64% felt they could not accurately assess wounds virtually and 55% felt that they would not want notifications intruding on them while they were away from work.

Only 62% of surgeons felt confident in a physiotherapist virtually assessing wounds, but 77% felt it would be suitable for an app-based physiotherapist service to initially assess patients with problems and escalate to a surgeon as required.

## Discussion

### Interpretation

This paper has established a consensus around several topics, including the management of surgical wounds, weight-bearing and orthotic boot use, while also identifying other areas requiring further consideration. Additionally, novel methods of follow-up and monitoring were explored and preferences for their use in a future rehabilitation intervention were established. The survey questions were intentionally designed to be polarising, aiming to achieve clear consensus where possible and identify areas of uncertainty for further research. Patient information can now reasonably incorporate these consensus-based recommendations, such as regarding bathing and scar massage timings. In contrast, sections exploring innovative monitoring methods and care delivery revealed more neutral or varied responses, emphasising the need for additional research in these areas.

Results from the HCP workshops and survey provide further credence to the concept that the surgical wound can be a visible early indicator for alerting patients to seek medical attention. Training patients to assess their wound, using descriptors as well as pictures of "expected" and "concerning" wounds could help both sides of their recovery: If their wound looks healthy, they can be reassured about continuing their rehabilitation, and if not, they will know to seek help, potentially sending a photo to a HCP in the first instance. Bruce et al. [30] found a fair correlation between photographic and in-person assessment for surgical site infection in a lower limb trauma population. Photographic assessment overestimated the surgical site infection rate compared to the Centre for Disease Control (CDC) criteria for surgical site infection (12% vs 6%). This indicates that remote photography is likely to be a safe screening tool for at-risk wounds, albeit not a tool to base treatment recommendations on.

The pathogenesis of persistent ankle swelling and pain after injury is poorly understood, and predicting which patients will experience it is difficult [31, 32]. Interventions to reduce ankle swelling and pain after injury have had limited success, and none has pervaded in routine clinical practice [33, 34]. The consensus achieved here should at least decrease the uncertainty and anxiety patients express around ankle swelling and give them the confidence to continue with their rehabilitation in the face of transient increases in pain and swelling.

The use of orthotic boots in the treatment of ankle fractures has been increasing, displacing the traditional dogma of immobilising patients in plaster casts for six to eight weeks [4, 35]. The recommendation to use the boot until the sixth post-operative week remains despite no evidence for this [2]. The six-week recommendation is likely a hangover from historical practice and also because some HCPs assume it is painful to weight-bear. Kortekangas et al. found that in non-operatively treated ankle fractures, three weeks of boot use was non-inferior to six weeks [5], and Smeeing et al. found that early weight-bearing without any boot support immediately post-operatively was superior to conventional treatment [36], but this strategy is rarely used in the UK [2]. A consensus was established that the orthotic boot is only for comfort and ease of walking, and assuming patients are permitted to weight-bear, it can be discarded as soon as the second post-operative week. This finding is not only important because it will allow people to forgo the orthotic boot sooner, but it means there is one less restriction on patients from HCPs and patients are one step closer to having autonomy over their rehabilitation. Previous qualitative research indicates that integrating these findings into clinical practice will likely require a multifaceted approach, with randomised controlled trials, clinical guidelines and peer influence serving as key drivers of change [19].

While there was agreement that once permitted to weight-bear, patients should do this gradually based on their own comfort, starting with axial loading, a consensus around what activities they should do while non-weight-bearing (e.g. putting their foot down for balance) was not achieved.

Metalwork removal is a controversial topic because unlike most other fractures, the historical practice had been that syndesmosis screws should be routinely removed two to three months post-operatively, due to the risk of screw breakage [38]. During the HCP survey, a consensus was found that patients do not require routine removal of metalwork after ankle fracture surgery, and unless they experience complications, surgeons are unlikely to offer removal before nine months post-operatively. Taken with the above considerations, patients can be reassured that they do not routinely require metalwork removal. The only caveat is that patients with broken and irritating syndesmosis screws could have them removed before nine months, as the syndesmosis will have healed, even if the bones have not.

The proposed rehabilitation app may make repeated surgical follow-up visits obsolete. Technology will facilitate remote patient clinical assessment, but as yet, it will not enable remote radiological assessment. Several studies have examined the necessity of repeat X-rays, beyond the intraoperative or two-week X-rays, finding that there was no change in alignment or that any changes were not clinically meaningful [39, 40]. However, a large (but non-consensus) proportion of surveyed HCPs wanted to see a six-week X-ray or signs of healing before discharging a patient from surgical follow-up. This is despite the fact that the mean time to radiographic healing is 82.2 days (SD 33.5) [39], well beyond even the 12-week follow-up visit. In contrast, 88% of HCPs would consider discharging patients from surgical follow-up after six weeks, though the same consensus was not achieved for discharge after two weeks.

### Strengths and limitations

Several studies have attempted to gain consensus around weight-bearing and other areas of ankle fracture rehabilitation, though none has succeeded [41, 42]. This is likely because they have used a purely quantitative approach and not sought to understand behavioural influences around weight-bearing decisions. The success of this paper in generating a consensus was likely attributable to the iterative, multistage generation of the survey statements.

The use of surveys for healthcare research has increased, particularly during the COVID-19 pandemic, and survey fatigue is a problem [43, 44]. The balance between the number of responses desired, the range of topics covered and the complexity of questions asked was carefully considered. As funding was available, range and complexity were prioritised. Furthermore, the proportion of respondents who were unable to comment on questions in their role was low and corresponded to physiotherapists being unable to comment on specific surgical questions. Guidance is sparse regarding the ideal sample size for consensus surveys, but considering the expert nature of respondents, the survey sample is similar to other healthcare research [45, 46].

## Conclusion

This paper has established a consensus for aspects of care around ankle fracture surgery rehabilitation. This will enable standardisation of advice around boot use, wound care and massage. Agreement around approaches to weight-bearing was established, reinforcing that "trusting your body" is the optimal way for patients to progress their rehabilitation, rather than waiting for HCPs approval or instructions. The design of a novel rehabilitation intervention has been considered and tailored to the desires of HCPs. Future research should focus on implementing and evaluating the proposed rehabilitation interventions within clinical settings to assess their feasibility and address challenges, particularly around the integration of digital platforms.

## Appendix 1

See (Table 2 and Table 3)

**Table 2:** Workshop participant sites (HCPs)

Site	N = 8
Oxford University Hospitals	4 (50%)
Queens Medical Centre Nottingham	1 (12.5%)
Royal Victoria Infirmary, Belfast	1 (12.5%)
Southampton General Hospital	1 (12.5%)
St George’s Hospital, London	1 (12.5%)

**Table 3:** Workshop participant characteristics (HCPs)

Participant ID	WAX Participant?	Gender	HCP Role	Subspecialty
1	No	Male	Physiotherapist	N/A
2	No	Male	Physiotherapist	N/A
3	No	Male	Consultant	Trauma
4	Yes	Male	Consultant	Trauma
5	Yes	Male	Physiotherapist	N/A
6	No	Male	Registrar	N/A
7	Yes	Female	Post-CCT Fellow	Foot and ankle
8	No	Male	Registrar	N/A

## Appendix 2

See Table 4

**Table 4:** Survey participant sites (HCPs)

Hospital Trust	N	Hospital Trust	N
Royal Victoria Infirmary, Belfast	9 (11%)	University Hospital Southampton	2 (2.5%)
Oxford University Hospital	7 (8.9%)	University Hospitals of Leicester	2 (2.5%)
East Surrey Hospital	4 (5.1%)	Altnagelvin Hospital	1 (1.3%)
East Sussex Hospitals	3 (3.8%)	Blackpool Teaching Hospitals	1 (1.3%)
Lancashire Teaching Hospitals	3 (3.8%)	Derriford Hospital	1 (1.3%)
Royal Cornwall Hospital	3 (3.8%)	Gloucestershire Hospitals	1 (1.3%)
Royal Preston Hospital	3 (3.8%)	Liverpool University Hospitals	1 (1.3%)
Salisbury NHS Foundation Trust	3 (3.8%)	Musgrave Park Hospital	1 (1.3%)
St George’s Hospital, London	3 (3.8%)	Northern General Hospital	1 (1.3%)
Birmingham University Hospitals	2 (2.5%)	Nottingham University Hospitals	1 (1.3%)
Cardiff and Vale Hospitals	2 (2.5%)	Royal Berkshire Hospital, Reading	1 (1.3%)
East Kent Hospitals	2 (2.5%)	Royal Derby Hospital	1 (1.3%)
Hampshire Hospitals	2 (2.5%)	Royal Devon & Exeter Hospital	1 (1.3%)
Maidstone & Tunbridge Wells Hospital	2 (2.5%)	Royal Sussex County Hospital	1 (1.3%)
Northern Care Alliance, Manchester	2 (2.5%)	St Mary’s Hospital, London	1 (1.3%)
Princess Alexandra Hospital, Harlow	2 (2.5%)	UK Chandler Hospital	1 (1.3%)
Queen Alexandra Hospital, Portsmouth	2 (2.5%)	University Hospital Coventry and Warwickshire	1 (1.3%)
Royal London Hospital	2 (2.5%)	William Harvey Hospital, Kent	1 (1.3%)
Royal United Hospital, Bath	2 (2.5%)	Unknown	1 (1.3%)
