# Correction: Co-design of a novel rehabilitation intervention for patients after ankle fracture surgery: establishing healthcare professional consensus

**DOI:** 10.1007/s12306-025-00898-6

**Published:** 2025-04-21

**Authors:** C. Bretherton, A. Al-Saadawi, H. Sandhu, J. Baird, X. Griffin

**Affiliations:** 1 Bone and Joint Health, Blizard Institute, Queen Mary University London, 4 Newark Street, London, E1 2AT UK; 2 Department of Trauma and Orthopaedic Surgery, Royal London Hospital, Barts Health NHS Trust, London, E1 1BB UK; 3 School of Medicine, Faculty of Medicine and Dentistry, Queen Mary University of London, London, UK; 4 Division of Health Sciences, Warwick Clinical Trials Unit, University of Warwick, Coventry, CV4 7AL UK; 5 Centre for Developmental Origins of Health and Disease, University of Southampton, Southampton, SO17 1BJ UK

**Correctionto: MUSCULOSKELETAL SURGERY** 10.1007/s12306-025-00881-1

In the original version of this article, the authors’ name H Sandhu, J Baird and X Griffin were incorrectly written as P H Sandhu P J Baird and P X Griffin.

